# From the Cochrane Library: Systemic Treatments for Metastatic Cutaneous Melanoma

**DOI:** 10.2196/30270

**Published:** 2021-09-23

**Authors:** Austin Hamp, Jarett Anderson, Torunn E Sivesind, Mindy D Szeto, Andreas Hadjinicolaou

**Affiliations:** 1 Arizona College of Osteopathic Medicine Midwestern University Glendale, AZ United States; 2 Department of Dermatology University of Colorado Anschutz Medical Campus Aurora, CO United States; 3 Human Immunology Unit, Institute of Molecular Medicine Radcliffe Department of Medicine University of Oxford Oxford United Kingdom; 4 Medical Research Council Cancer Unit Hutchison/MRC Research Centre University of Cambridge, Cambridge Biomedical Campus Cambridge United Kingdom

**Keywords:** melanoma, metastatic melanoma, skin neoplasms, angiogenesis inhibitors, monoclonal antibodies, antineoplastic agents, Cochrane reviews, systematic reviews, clinical trials, randomized controlled trials, dermatology

Melanoma is the most lethal type of skin cancer, with a 5-year survival rate of only 22.5% for stage IV (metastatic) disease [1]. Furthermore, with its steadily increasing incidence rate of 5% to 7% per year predicted through 2031, melanoma represents a significant health burden in the United States [1]. Treatment options for metastatic melanoma have changed dramatically with novel therapeutic strategies. However, a consensus on treatment and quality of evidence has yet to be established. “Systemic treatments for metastatic cutaneous melanoma,” a 2018 Cochrane review, assessed the beneficial and harmful effects of these new classes of drugs in treating unresectable metastatic melanoma, defined as stage IIIC or stage IV [2].

This review found high-quality evidence that many newer agents, such as immune checkpoint inhibitors and targeted therapies in the form of small-molecule inhibitors, were more effective than conventional chemotherapies (ie, dacarbazine and temozolomide) in treating unresectable metastatic melanoma. Table 1 summarizes significant findings of the Cochrane review on drug comparisons.

As noted in Table 1, BRAF inhibitors and BRAF inhibitors + mitogen-activated protein kinase (MAPK; MEK) inhibitors (both are MAPK pathway inhibitors) provide improved survival for patients with metastatic melanoma with BRAF gene mutations. These treatment options are of particular importance, as 40% to 60% of metastatic melanomas harbor the BRAF mutation [3]. A 2021 meta-analysis supported the findings of this Cochrane review, concluding improved overall survival (hazard ratio [HR] 0.59, 95% CI 0.47-0.74) and progression-free survival (HR 0.24, 95% CI 0.19-0.3) when comparing BRAF + MEK inhibitors against conventional chemotherapy for unresectable metastatic melanoma (TNM [tumor, node, metastasis] stage IIIc) [3]. While these data are encouraging, additional randomized controlled studies are warranted to further elucidate outcome differences between these combination treatment strategies.

**Table 1:** A Cochrane review of metastatic melanoma therapies for overall survival, progression-free survival, and toxicity rate.

Drug therapy comparison		Overall survival	Progression-free survival^a^	Toxicity rate^b^
**Antiprogrammed cell death protein 1 (anti-PD1) vs conventional chemotherapy^c^**				
	Outcome	Improved	Improved	Decreased
^ ^	Corresponding risk^d^ vs assumed risk^e^	320 (95% CI 290-360) deaths per 1000 vs 600 deaths per 1000, respectively	610 (95% CI 520-690) per 1000 vs 850 per 1000, respectively	165 (95% CI 93-291) toxicities per 1000 vs 300 per 1000, respectively
^ ^	Relative effect	HR^f^ 0.42, 95% CI 0.37-0.48, 1 study, N=418	HR 0.49, 95% CI 0.39-0.61, 2 studies, N=957	RR^g^ 0.55, 95% CI 0.31-0.97, 3 studies, N=1360
^ ^	Evidence quality^h^	High	Moderate	Low
**Anti-PD1 vs anticytotoxic T-lymphocyte–associated protein 4 (anti-CTLA4)**				
	Outcome	Improved	Improved	Decreased
	Corresponding risk vs assumed risk^e^	428 (95% CI 423-454) deaths per 1000 vs 600 deaths per 1000, respectively	641 (95% CI 612-679) per 1000 vs 850 per 1000, respectively	278 (95% CI 215-362) toxicities per 1000 vs 398 per 1000, respectively
	Relative effect	HR 0.63, 95% CI 0.60-0.66, 1 study, N=764	HR 0.54, 95% CI 0.50-0.60, 2 studies, N=1465	RR 0.70, 95% CI 0.54-0.91, 2 studies, N=1465
	Evidence quality	High	High	Low
**Anti-PD1 and anti-CTLA4 vs anti-CTLA4 alone**				
	Outcome	—^i^	Improved	No significant difference
	Corresponding risk vs assumed risk^j^	—	425 (95% CI 375-478) per 1000 vs 750 per 1000, respectively	278 (95% CI 215-362) toxicities per 1000 vs 398 per 1000, respectively
	Relative effect	—	HR 0.40, 95% CI 0.35-0.46, 2 studies, N=738	RR 1.57, 95% CI 0.85-2.92, 2 studies, N=764
	Evidence quality	—	High	Low
**BRAF inhibitors vs conventional chemotherapy^c^**				
	Outcome	Improved	Improved	No significant difference
	Corresponding risk vs assumed risk^e^	307 (95% CI 226-407) deaths per 1000 vs 600 deaths per 1000, respectively	401 (95% CI 328-475) per 1000 vs 600 per 1000, respectively	433 (95% CI 163-1135) toxicities per 1000 vs 341 toxicities per 1000, respectively
	Relative effect	HR 0.40, 95% CI 0.28-0.57, 2 studies, N=925	HR 0.27, 95% CI 0.21-0.31, 2 studies, N=925	RR 1.27, 95% CI 0.48-3.33, 2 studies, N=408
	Evidence quality	High	High	Low
**Mitogen-activated protein kinase (MEK) inhibitors vs conventional chemotherapy^c^**				
	Outcome	No significant difference	Improved	Increased
	Corresponding risk vs assumed risk^e^	541 (95% CI 412-682) deaths per 1000 vs 600 deaths per 1000, respectively	667 (95% CI 549-781) per 1000 vs 850 per 1000, respectively	665 (95% CI 446-995) toxicities per 1000 vs 413 toxicities per 1000, respectively
	Relative effect	HR 0.85, 95% CI 0.58-1.25, 3 studies, N=496	HR 0.58, 95% CI 0.42-0.80, 3 studies, N=496	RR 1.61, 95% CI 1.08-2.41, 1 study, N=91
	Evidence quality	Low	Moderate	Moderate
**BRAF inhibitors + MEK inhibitors vs BRAF inhibitors alone**				
	Outcome	Improved	Improved	Increased
	Corresponding risk vs assumed risk^k^	260 (95% CI 204-321) deaths per 1000 vs 350 deaths per 1000, respectively	490 (95% CI 411-574) per 1000 vs 700 per 1000, respectively	500 (95% CI 421-594) toxicities per 1000 vs 495 toxicities per 1000, respectively
	Relative effect	HR 0.70, 95% CI 0.59-0.82, 4 studies, N=1784	HR 0.56, 95% CI 0.44-0.71, 4 studies, N=1784	RR 1.01, 95% CI 0.85-1.20, 4 studies, N=1774
	Evidence quality	High	Moderate	Moderate
**Chemotherapy + antiangiogenic drugs^l^ vs conventional chemotherapy^c^**				
	Outcome	Improved	Improved	No significant difference
	Corresponding risk vs assumed risk^e^	423 (95% CI 338-524) deaths per 1000 vs 600 deaths per 1000, respectively	730 (95% CI 627-825) per 1000 vs 850 per 1000, respectively	185 (95% CI 25-1447) toxicities per 1000 vs 272 toxicities per 1000, respectively
	Relative effect	HR 0.60, 95% CI 0.45-0.81, 2 studies, N=324	HR 0.69, 95% CI 0.52-0.92, 2 studies, N=324	RR 0.68, 95% CI 0.09-5.32, 2 studies, N=324
	Evidence quality	Moderate	Moderate	Low
**Polychemotherapy^m^ vs conventional chemotherapy^c^**				
	Outcome	None	None	Increased
	Corresponding risk vs assumed risk^e^	No significant difference	No significant difference	372 (95% CI 272-512) toxicities per 1000 vs 189 toxicities per 1000, respectively
	Relative effect	HR 0.99, 95% CI 0.85-1.16, 6 studies, N=594	HR 1.07, 95% CI 0.91-1.25, 5 studies, N=398	RR 1.97, 95% CI 1.44- 2.71, 3 studies, N=390 ** **
	Evidence quality	High	High	Moderate

^a^Progression-free survival is defined as the time from randomization until diagnosis of disease recurrence (local or distant/metastatic). The numbers listed refer to event rates (death rates and progression rates) [2].

^b^Toxicity is defined as the occurrence of grade 3 or higher adverse events according to the World Health Organization scale.

^c^Dacarbazine and its orally available derivative, temozolomide, both of which cross-link DNA, inhibiting transcription and replication [2].

^d^Corresponding risk is based on the assumed risk in the comparison group and the relative effect of the intervention.

^e^Assumed risk (which is defined as the median control group risk across all studies): 1-year overall survival rate (40%); assumed risk in the control population: 1-year progression-free survival rate (15%); assumed risk in the control population: toxicity rate across the control arms of the included trials.

^f^HR: hazard ratio.

^g^RR: risk ratio.

^h^High-quality evidence: further research is very unlikely to change the confidence in the estimate of effect; moderate-quality evidence: further research is likely to have an important impact on the confidence in the estimate of effect and may change the estimate; low-quality evidence: further research is very likely to have an important impact on the confidence in the estimate of effect and is likely to change the estimate; very low-quality evidence: very uncertain about the estimate.

^i^No data available.

^j^Assumed risk in the control population: 1-year progression-free survival rate (15%); assumed risk in the control population: toxicity rate across the control arms of the included trials.

^k^Assumed risk in the control population: 1-year overall survival rate (65%); assumed risk in the control population: 1-year progression-free survival rate (30%); assumed risk in the control population: toxicity rate across the control arms of the included trials.

^l^Bevacizumab and endostar.

^m^Dacarbazine in combination with other chemotherapeutics.

Despite the efficacy of BRAF + MEK inhibitors in treating BRAF-mutated melanoma, about 20% of BRAF-mutated melanomas demonstrate resistance to this therapy [4]. Therefore, the pursuit of alternative treatments is necessary. New therapies, such as T-cell therapies, which include tumor-infiltrating lymphocytes (TILs), T-cell receptor therapy, and chimeric antigen receptor T-cell therapy, have shown promising results in treating metastatic melanoma. A recent study reported an objective response rate of 36% (95% CI 25%-49%) and a median duration of response that was not reached after an 18.7-month median follow-up (range 0.2-34.1 months) in patients with metastatic melanoma (stage IIIc or IV) treated with TILs [5]. These therapies present an exciting new avenue to treating metastatic melanoma in patients who have not responded to approved therapy, as there remain very few treatments to improve outcomes in these patients. Additional studies are underway to determine the efficacy of these T-cell therapies on metastatic melanoma and assess the duration of response.

In conclusion, this Cochrane review provides convincing evidence supporting the use of new therapeutics compared to chemotherapy alone. Given recent evidence of resistance to older drugs, there is an ongoing and urgent need for alternative treatment options and approaches [4]. We encourage additional study and evaluation of evidence regarding novel therapies to accurately and comprehensively identify the most effective treatments for metastatic melanoma, especially the individualized treatment of specific melanoma subsets.

## Abbreviations

HR: hazard ratio
MAPK: mitogen-activated protein kinase
TIL: tumor-infiltrating lymphocyte
TNM: tumor, node, metastasis
